# Homegarden agroecosystems managed by Salar people on Qinghai-Tibet Plateau

**DOI:** 10.1186/s13002-021-00448-x

**Published:** 2021-03-23

**Authors:** Mingjing Zhu, Binsheng Luo, Ben La, Ruijie Chen, Fenggui Liu, Chunlin Long

**Affiliations:** 1 College of Geographical Sciences, Qinghai Normal University, Xining, 810008 China; 2 College of Life and Environmental Sciences, Minzu University of China, Beijing, 100081 China; 3 Kunming Institute of Botany, Chinese Academy of Sciences, Kunming, 650201 China

**Keywords:** Homegarden, Traditional culture, Agrobiodiversity, Ecosystem services, Salar people

## Abstract

**Background:**

Salar is a Turkic-speaking Islamic ethnic group in China living mainly in Xunhua Salar Autonomous County (Xunhua or Xunhua County), Qinghai-Tibet Plateau. Salar people are skilled in horticulture and their homegarden (HG) management. They are regarded as the first people on the Qinghai-Tibet Plateau to practice horticulture, especially manage their HGs, traditional farming systems, and supplementary food production systems. Traditional knowledge of Salar people associated with their HGs always contributes significantly to the local livelihood, food security, ornamental value, and biodiversity conservation. The cultivation of different plants in HGs for self-sufficiency has a long tradition in China’s rural areas, especially in some mountainous areas. However, Salar traditional HGs have not been described. The present paper aims to report the features of Salar HGs mostly based on agrobiodiversity and its ecosystem services.

**Methods:**

The methods used in this work included semi-structured interviews and participatory observation. A total of 60 households in three townships, 9 villages were surveyed. There are 4–12 family members in each household, aged from 20 to 86 years old. The homestead size is between 200 and 1200 m^2^. Plant species cultivated in Salar HGs were identified according to *Flora of China*. Based on a comprehensive survey of Salar HGs and related to background data, we identified and characterized the most important services and functions provided by Salar HGs.

**Results:**

According to primary production systems, there are 4 different types of Salar HGs, including ornamental focus, product focus, dual-purpose and multi-purpose. In total, 108 (excluding weeds and bonsai) plant species were recorded in Salar HGs, within 43 plant families. The most important and frequently used plants are *Rosa chinensis*, *Armeniaca vulgar*, *Prunus salicina*, and *Ziziphus jujuba.* About 4 to 32 plant species were recorded in each homegarden. We found that the Salar HGs, as a typical agroecosyste, prossess multiple servcices and functions that directly benefit households according to the field investigation.

**Conclusion:**

This paper reveals the floristic diversity of Salar HGs. It presents useful information in the homegarden agroecosystem of Salar people, such as HG types and species diversity in Salar HGs. Ecosystem functions and services research suggested that the Salar HG agroecosystem provides agroecosystem services mainly related to supply and culture services. Salar HGs are important as food supplement resources, aesthetics symbol, and cultural spaces.

## Background

Salar people are Turkic-speaking Islamic people as an ethnic group with a small population in China. Salar people live primarily in Xunhua Salar Autonomous County, Qinghai Province, Northwest China (Xunhua or Xunhua County thereafter). According to a China National Institute of Statistics report, approximately 140,000 Salar people live in China [1]. Their origins are uncertain, but Salar people consider that their ancestors left Samarkand (in present-day Uzbekistan) during the thirteenth century and eventually settled in the present location [2]. They still speak the language of their ancestors but without a writing system. Salar people are regarded as the first people in the Qinghai-Tibet plateau to practice horticulture [2]. Thus, their traditional culture has been well-known for traditional home architecture and homegarden (HG) management. The Salar HGs take a significant place in Salar culture, bringing them a great sense of pride.

HG researches have mainly involved agriculture, ecology, nutrition, and biodiversity, especially ethnobotanical documentation of cultivated plants [3–7]. Most studies described HGs as a traditional farming system, supplementary food production system, and one of the oldest land-use systems [8–10]. The researches about HGs emphasized plant diversity, multiple functions, and other benefits for peasant farmers [11–13]. Those HGs are featured of highly diverse cultivated plants, individual houses, and livestock, and they are also regarded as a typical sustainable agricultural production system [14–16]. As an essential component, the diverse plant species within HGs are used as food, spices, stimulants, medicines, beverages, fodder, and shelter [17]. Some recent studies on agroforestry and traditional production systems have begun to consider the capacity of HGs for adapting to the ecosystem and climate change-related challenges [18].

HGs bridge the social and natural environment, linking with cultivated species and natural ecosystems, conserving the genetic diversity [19]. They were reported to provide multi-diets and nutritional improvement for low-income groups [20]. However, the increasing human population, urbanization, and environmental stress have aggravated the breakdown of those traditional agroecosystems, including the traditional HGs [21].

HGs are distributed worldwide and contribute to the functioning and sustainability of the larger agroecosystem [22, 23] but are predominantly a tropical phenomenon. Little studies have focused on the Qinghai-Tibet Plateau, where HGs are an essential source of production and *in situ* conservation of biodiversity. Historically, Salar people have a tradition of operating orchards and managing their HGs in the small and narrow Yellow River valley with a relatively warm climate and fertile soil. Salar HGs are ideal for studying traditional agroecosystems and related traditional knowledge in Qinghai-Tibet Plateau. However, the Salar people and their traditional agriculture had not been reported in any academic literature.

This study analyzed the structure and multiple functions of the Salar HGs agroecosystem based on the ethnobotanical interview on 60 local households. Through this research, we attempt to contribute some scientific references and suggestions for policy-making. This study may also help for agrobiodiversity conservation at a community level and positively influence people’s management, protection, and inheritance of HG agroecosystem to achieve sustainable development.

## Methods

### Study area

**Fig. 1:**
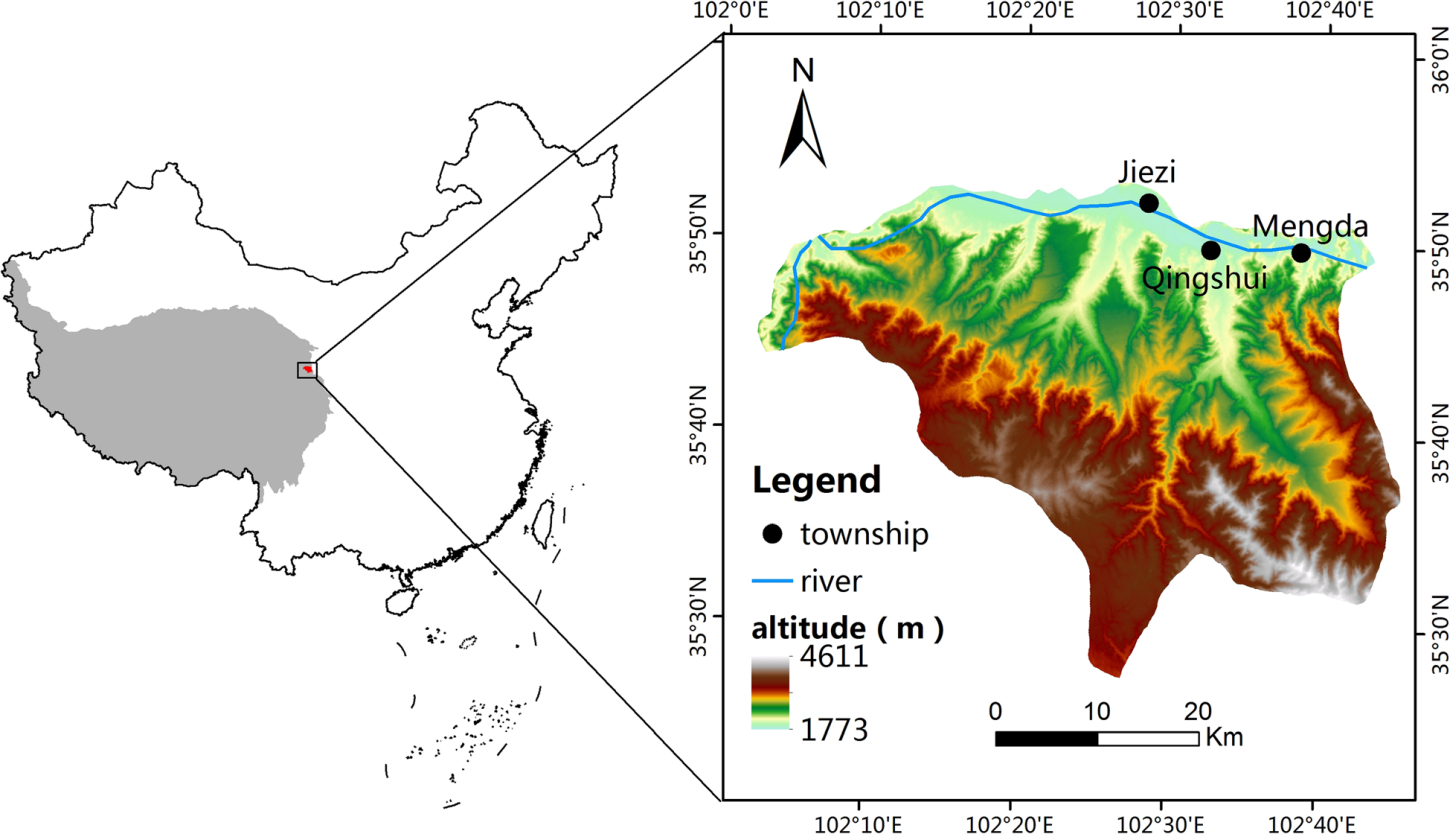
Geographical location of the study area

The field investigations were conducted on Eastern Qinghai-Tibet Plateau (between 102° 1' E–102° 7' E and 35° 4' N–35° 8' N) and located in the east of the Yellow River valley area where the average annual temperature and rainfall are about 8.5 °C and 264.4 mm, respectively and an average altitude of 2300 m, a relatively warm climate for crop growth. It is one of the areas that the earliest human activities in the Yellow River basin [2]. The area is characterized by ecological and cultural richness. Nine villages (Fig. 1) were selected as the investigation sites because they mainly live in these villages and are known for widespread HGs and rich management experience.

### Data collection

In the preparatory phase, the knowledge framework was constructed based on the literature review, mainly including the documentations about the Salar history and culture and their homegarden management traditions.

The next step was the data collection through field observation and ethnobotanical interviews on local informants [24]. Several field investigations have been carried out during 2018 and 2019. Forming elements, structures, plant composition, and cultural activities of Salar HGs were recorded during the field observation [25]. Semi-structured interviews were conducted to obtain information about the Salar HGs. The questions we asked mainly included

1) What is this plant you grow?

2) What is this plant for?

3) What do you think is the primary function/functions of the HG for your family?

### Homegarden selection

The selection of HGs for studying was random. Some corner cases were excluded, such as HGs with less than 100 m^2^, those not productive for most of the year, only bonsais in the HGs, or HGs not owned by the Salar people. The households of the selected HGs are also the participants of our ethnobotanical interviews. The managers of the Salar HGs who have a better experience of gardening are considered as the key informants of the study. In total, 60 households, including 120 Salar people, were interviewed face-to-face. Of these 120 participants, 65 people are the managers of Salar HGs, and the rest of the participants are randomly met and interviewed in the field or the street. The Salar HG managers’ sociodemographic information was also collected, including the age, gender, education level, gardening history, and residency length in the village (Table 1).

**Table 1:** Socioeconomic characteristics of Salar HG managers

Socio-economic characteristics		Number (%) of respondents
Sex	Male	6 (9.2)
	Female	59 (90.8)
Age	20–35	8 (12.3)
	36–50	32 (49.2)
	51–65	13 (20.0)
	66–86	12 (18.4)
Literacy status	Literate Illiterate	15 (23.1) 50 (76.9)
	< 4000	14 (23.3)
Annual income ($)	4000–1500	31 (51.7)
	> 1500	15 (25.0)
Family member	3–6 7–9 10–12	37 (61.6) 17 (28.3) 5 (10.1)
History of homegarden	10–35	25 (41.6)
	36–55	20 (33.3)
	56–90	15 (25.1)
Homestead size (m^2^)	200–400	32 (53.3)
	410–660	20 (33.3)
	670–1200	8 (13.4)
	< 50	35 (58.3)
Cultivated area (m^2^)	50–300	20 (33.3)
	> 310	15 (8.4)

### Agrobiodiversity census

An ethnobotanical inventory of the cultivated plants in Salar HGs is finished with the assistances of Salar participants. The plants recorded are usually used for different purposes like food, ornamentals, dye materials, and medicines. Voucher specimens were collected in Salar HGs, identified by botanists scientifically based on the *flora of China,* and saved in the Minzu University of China.

### Cataloging functions and ecosystem services

Identification and characterization of agroecosystem services and functions of Salar HGs were analyzed based on information obtained from the literature review, participant and non-participant observation, and semi-structured interviews [26]. We used participatory and non-participatory observation methods to comprehend the Salar HG structure during fieldwork and reveal the relationship among the Salar HGs, local community, and traditional Salar culture [27]. We also observed the homegarden work was performed by Salar people to understand the possible homegarden functions and agroecosystem services comprehensively [28–33].

## Results

### The analysis of the participants

We have recorded the information about the HG managers in Table 1. As we can see, the female is the dominant gender of Salar HG managers. The woman is the leading laborer for farming work in the field; comparatively, the works in Salar HGs require less strength. More than 50% of HG managers are younger than 50 years old, meaning the traditional knowledge about HG practice is inherited well among generations. A great majority of HG managers have not accepted any education; they absorbed the knowledge about HG, usually from the last generation and actual practices. About the families’ income (not just from the HGs), about 1/4 families earned less than 4000 dollars each year, and 1/4 families can earn more than 15,000 dollars. One-half of the families can earn a middle level, 4000–15,000 dollars. The history, the size, and the planting area of Salar HGs are also recorded in Table 1. Usually, a bigger HG goes with a bigger planting area.

### Salar HG management

According to an orally spread legend, Salar ancestors migrated from Central Asia to Qinghai-Tibet Plateau—" they rode camels and brought the seeds of wheat."—which might indicate that their ancestors were engaged in the planting industry when they lived in Central Asia. Until now, the livelihood of Salar people is still mainly based on the planting industry.

Salar HGs are called “Bahe” locally, which means “a fenced place for growing flowers and trees”. The Salar people are the first ethnic group to practice the horticulture industry on the Qinghai-Tibet Plateau and have inherited it for several generations. For Salar households, the oldest females in the family are responsible for managing HGs because it requires less time and labor than major farming activities. Males have been increasingly engaging in the small business or going out for work. Traditional management practices for soil fertility were found in some HGs, including composting human wastes, livestock manure, and farmyard manure. Intercropping in some HGs was also a solution to maintain soil fertility.

### Agrobiodiversity in HGs

**Fig. 2:**
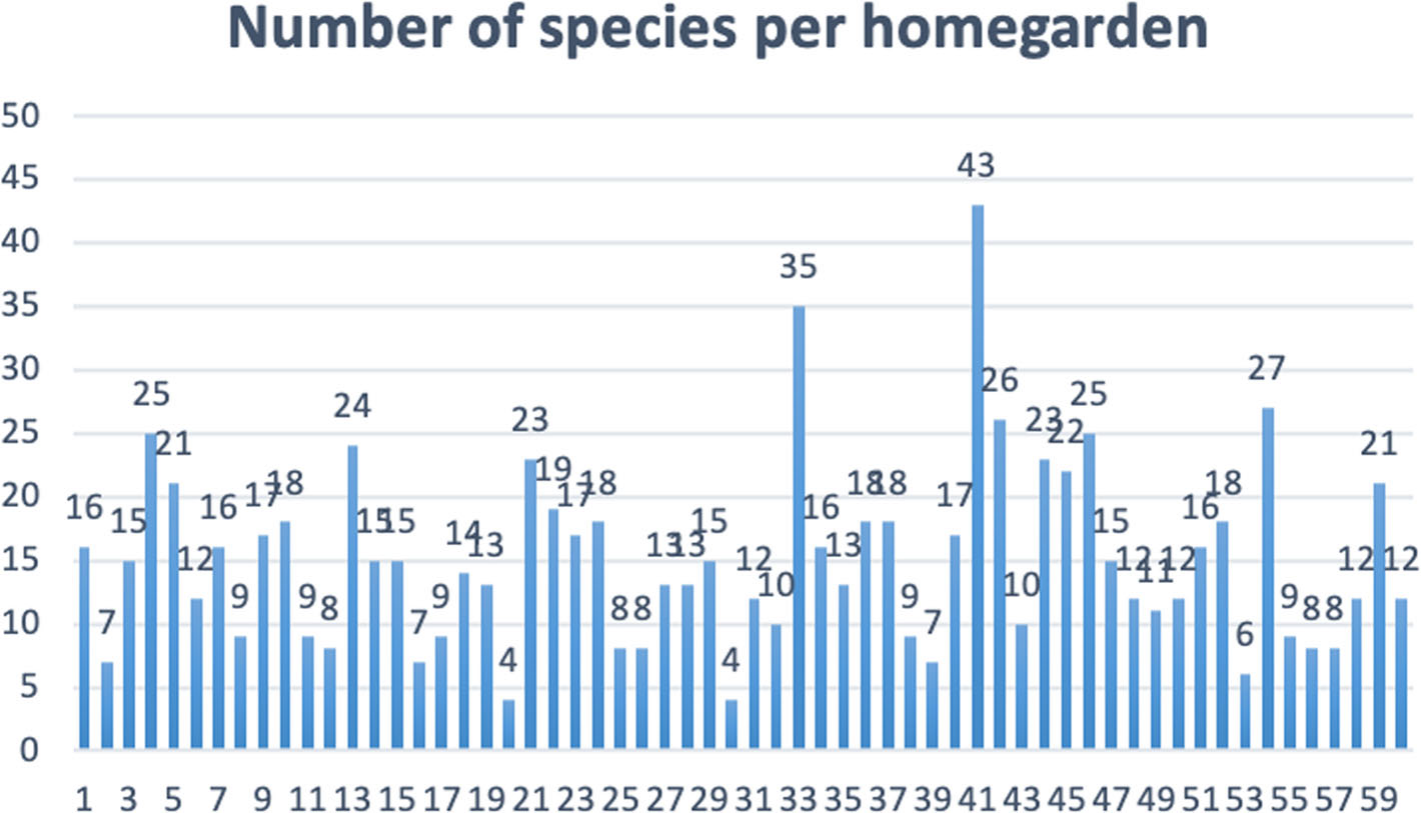
Number of species per homegarden

HGs are recognized worldwide as sustainable agroecosystems that are good repositories of genetic resources [34–38]. During our field investigation, in total, 108 (excluding weeds and bonsai) plant species were recorded within 43 families. Four to 43 species were found in each HG (Fig. 2). The 60 surveyed gardens contained 42 ornamental species (Table 2), 27 vegetable species (Table 3), 24 fruit species (Table 4), and species for other purposes (Table 5). Ornamentals were the most species-rich in the Uses category, followed by vegetables, fruits, and 15 species for other purposes, showing great biodiversity in Salar HGs.

**Table 2:** Ornamental species recorded and used in Salar HGs

No.	Scientific name	Family	Uses
1	*Acorus calamus*	Acoraceae	Ornamental
2	*Alcea rosea* L.	Malvaceae	Ornamental
3	*Amygdalus triloba* (Lindl.) Ricker	Rosaceae	Ornamental
4	*Belamcanda chinensis* (L.) Redouté	Iridaceae	Ornamental
5	*Bougainvillea glabra* Choisy	Nyctaginaceae	Ornamental
6	*Calendula officinalis* L.	Compositae	Ornamental
7	*Callistephus chinensis* (L.) Nees	Compositae	Ornamental
8	*Canna indica* L.	Cannaceae	Ornamental
9	*Celosia cristata* L.	Amaranthaceae	Ornamental, experimental
10	*Chrysanthemum morifolium* Ramat.	Compositae	Ornamental
11	*Cosmos bipinnata* Cav.	Compositae	Ornamental
12	*Dahlia pinnata Cav.*	Compositae	Ornamental
13	*Dicentra spectabilis* (L.) Lem.	Papaveraceae	Ornamental
14	*Eschscholzia californica* Cham.	Papaveraceae	Ornamental
15	*Fuchsia hybrida* Hort. ex Sieb. et Voss.	Onagraceae	Ornamental
16	*Helianthus annuus* L.	Compositae	Ornamental, snacks
17	*Hemerocallis fulva* (L.) L.	Liliaceae	Ornamental
18	*Hosta plantaginea* (Lam.) Aschers.	Liliaceae	Ornamental
19	*Ipomoea nil* (L.) Roth	Convolvulaceae	Ornamental
20	*Iris tectorum* Maxim.	Iridaceae	Ornamental
21	*Lilium brownii* var*. viridulum* Baker.	Liliaceae	Ornamental
22	*Lilium pumilum* DC.	Liliaceae	Ornamental
23	*Lilium tigrinum* Ker-Gawl.	Liliaceae	Ornamental
24	*Nerium oleander* L.	Apocynaceae	Ornamental
25	*Osmanthus fragrans* (Thunb.) Lour.	Oleaceae	Ornamental, perfume
26	*Paeonia anomala* L. subsp. *veitchii* (Lynch) D. Y. Hong et K. Y. Pan	Paeoniaceae	Ornamental
27	*Paeonia lactiflora* Pall.	Ranunculaceae	Ornamental
28	*Paeonia suffruticosa* Andr.	Ranunculaceae	Ornamental
29	*Pelargonium hortorum* Bailey	Ranunculaceae	Ornamental
30	*Rosa chinensis* Jacq.	Rosaceae	Ornamental
31	*Rosa multiflora* Thunb.	Rosaceae	Ornamental
32	*Rosa multiflora* Thunb. var. *carnea* Thory	Rosaceae	Ornamental
33	*Rosa rugosa* Thunb.	Rosaceae	Ornamental
34	*Rosa xanthina* Lindl.	Rosaceae	Ornamental
35	*Rudbeckia laciniata* L.	Compositae	Ornamental
36	*Salvia splendens* Ker-Gawl.	Labiatae	Ornamental
37	*Sorbaria sorbifolia* (L.) A. Br.	Rosaceae	Ornamental
38	*Syringa reticulata subsp. amurensis* (Rupr.) P. S. Green et M. C. Chang.	Oleaceae	Ornamental
39	*Tagetes erecta* L.	Compositae	Ornamental
40	*Tropaeolum majus* L.	Tropaeolaceae	Ornamental
41	*Viola tricolor* L.	Violaceae	Ornamental
42	*Zinnia elegans* Jacq.	Compositae	Ornamental

**Table 3:** Fruits recorded and used in Salar HGs

No.	Scientific name	Family	Uses
1	*Amygdalus persica* L.	Rosaceae	Fruit, shade
2	*Armeniaca vulgaris* Lam.	Rosaceae	Fruit, shade
3	*Cerasus pseudocerasus* (Lindl.) G. Don	Rosaceae	Fruit
4	*Chaenomeles sinensis* (Thouin) Koehne	Rosaceae	Experimental
5	*Citrullus lanatus* (Thunb.) Matsum. et Nakai	Cucurbitaceae	Fruit
6	*Citrus reticulata* Blanco	Rutaceae	Fruit, ornamental
7	*Crataegus pinnatifida* Bge.	Rosaceae	Fruit, shade
8	*Diospyros kaki* Thunb.	Ebenaceae	Fruit, shade
9	*Elaeagnus angustifolia* L.	Elaeagnaceae	Fruit, perfume
10	*Eriobotrya japonica* (Thunb.) Lindl.	Rosaceae	Experimental
11	*Ficus carica* L.	Moraceae	Dry fruit
12	*Fragaria × ananassa* Duch.	Rosaceae	Fruit
13	*Juglans regia* L.	Juglandaceae	Dry fruit, shade
14	*Malus pumila* Mill.	Rosacea	Fruit
15	*Morus alba* L.	Moraceae	Fruit
16	*Prunus cerasifera* Ehrhar f. *atropurpurea* (Jacq.) Rehd.	Rosacea	Fruit, shade
17	*Prunus domestica* L.	Rosaceae	Fruit
18	*Prunus persica* var*. nectarina* Maxim.	Rosaceae	Fruit
19	*Prunus salicina* Lindl.	Rosaceae	Fruit
20	*Punica granatum* L.	Punicaceae	Experimental, fruit
21	*Pyrus ussuriensis* Maxim.	Rosaceae	Fruit
22	*Sorbus alnifolia* (Sieb. et Zucc.) K. Koch	Rosaceae	Fruit, shade
23	*Vitis vinifera* L.	Vitaceae	Fruit, ornamental
24	*Ziziphus jujuba* Mill.	Rhamnaceae	Fruit

**Table 4:** Vegetables recorded and used in Salar HGs

No.	Scientific name	Family	Uses
1	*Allium fistulosum* L.	Liliaceae	Vegetable, spice
2	*Allium tuberosum* Roxb.	Liliaceae	Vegetable, spice
3	*Apium graveolens* L.	Umbelliferae	Vegetable, medicinal
4	*Beta vulgaris* L.	Chenopodiaceae	Vegetable, spice
5	*Brassica chinensis* L. var. *oleifera* Makino et Namot	Cruciferae	Vegetable
6	*Brassica oleracea* L.	Cruciferae	Vegetable
7	*Brassica oleracea* var. *botrytis* L.	Cruciferae	Vegetable
8	*Capsicum annuum* L.	Solanaceae	Vegetable, spice
9	*Chrysanthemum coronarium* L.	Compositae	Vegetable
10	*Cichorium endivia* L.	Compositae	Vegetable
11	*Coriandrum sativum* L.	Umbelliferae	Vegetable, spice
12	*Cucumis sativus* L.	Cucurbitaceae	Vegetable, ornamental
13	*Cucurbita moschata* (Duch. ex Lam.) Duch. ex Poiret	Cucurbitaceae	Vegetable
14	*Cucurbita pepo* L.	Cucurbitaceae	Vegetable
15	*Lactuca sativa* L. var. *ramosa* Hort.	Compositae	Vegetable
16	*Lactuca sativa* var. *longifolia* Lam	Compositae	Vegetable
17	*Luffa cylindrica* (L.) Roem.	Cucurbitaceae	Vegetable
18	*Lycopersicon esculentum* Mill.	Solanaceae	Vegetable
19	*Phaseolus vulgaris* L.	Leguminosae	Vegetable
20	*Pisum sativum* L.	Leguminosae	Vegetable
21	*Raphanus sativus* L.	Cruciferae	Vegetable
22	*Solanum melongena* L.	Solanaceae	Vegetable
23	*Spinacia oleracea* L.	Chenopodiaceae	Vegetable
24	*Vicia faba* L.	Leguminosae	Vegetable
25	*Vigna unguiculata* (L.) Walp.	Leguminosae	Vegetable
26	*Zanthoxylum bungeanum* Maxim.	Rutaceae	Spice
27	*Zea mays* L.	Gramineae	Seed

**Table 5:** Other useful species recorded in Salar HGs

No.	Scientific name	Family	Uses
1	*Artemisia argyi* Levl. et Van.	Compositae	Medicinal
2	*Buxus sinica* (Rehd. et Wils.) Cheng subsp. *sinica* var. *parvifolia* M. Cheng	Buxaceae	Ornamental
3	*Eucommia ulmoides* Oliver	Eucommiaceae	Medicinal
4	*Euonymus japonicus* Thunb. var. *aurea-marginatus* Hort.	Celastraceae	Ornamental
5	*Gynostemma pentaphyllum* (Thunb.) Makino	Cucurbitaceae	Experimental, medicinal
6	*Hordeum distichon* var. nudum L.	Gramineae	Seed
7	*Impatiens balsamina* L.	Balsaminaceae	Dye
8	*Mukdenia rossii* (Oliv.) Koidz.	Saxifragaceae	Ornamental
9	*Opuntia stricta* (Haw.) Haw. var. *dillenii* (Ker-Gawl.) Benson	Cactaceae	Medicinal
10	*Paulownia tomentosa* (Thunb.) Steud. var. *tsinlingensis* (Pai) Gong Tong	Scrophulariaceae	Shade
11	*Picea crassifolia* Kom.	Pinaceae	Shade
12	*Platycladus orientalis* (L.) Franco	Cupressaceae	Ornamental
13	*Potentilla glabra* Lodd.	Rosaceae	Ornamental
14	*Robinia pseudoacacia* L.	Leguminosae	Shade, ornamental
15	*Trigonella foenum-graecum* L.	Leguminosae	Dye, Spice

We listed Top 5 plant species with the highest RF values in Table 6. The most popular one is *Rosa chinensis*, with the highest RFC value. *Rosa chinensis* is one of the most popular flowering plants worldwide with a long flowering phase and beautiful shape and is very easy to grow. Salar people love flowers, that is the reason they grow this species so frequently. We found not only the commercial varieties but also some traditional ones grown in Salar HGs. The following popular species are *Armeniaca vulgar*, *Prunus salicina*, and *Ziziphus jujuba*. Those fruits can fit the local climate, where the diurnal temperature variation is significant and gives the fruits a better sweet taste. *Ziziphus jujuba* was also described in *the Koran*: The prophet encourages people to grow *Ziziphus jujuba* to deal with the end of the world. Thus, this species has a special position in Salar culture. *Lycopersicon esculentum* is also popular locally. It is easy to grow and contains important nutrients like Vitamin C and Vitamin D to supplement human needs. Tomato is one of the favorite foods of Salar people, and they usually use it as a fundamental ingredient when cooking wheaten food (Salar pasta).

**Table 6:** Top 5 plant species with the highest RF value

Species	Occurrence No.	Relative frequency (rf)
*Rosa chinensis* Jacq.	56	93.3%
*Armeniaca vulgaris* Lam.	49	81.7%
*Prunus salicina* Lindl.	45	75.0%
*Ziziphus jujuba* Mill.	38	63.3%
*Lycopersicon esculentum* Mill.	35	58.3%

As we mentioned, the Salar people like ornamental plants as the decoration of HGs. Thus, bonsais become one of the essential parts of the plant diversity of Salar HGs. However, regular ornamental plants cannot be grown in this area for generations because of the plateau environment. Therefore, almost every household in Salar communities purchases bonsais as decorations from the markets. During the investigation, we recorded the most common bonsai in Salar HGs including *Bougainvillea spectabilis*, *Pelargonium hortorum*, *Fuchsia hybrida*, *Hydrangea macrophylla*, and *Begonia cucullata* var. *hookeri*.

We also found that plant exchanges between Salar farmers has contributed significantly to the plant diversity in Salar HGs. Planting materials, including the seeds, roots, shoots, etc., were usually primarily inherited from family members. However, they also love to exchange plant materials for growing, which is much more than purchasing from the markets. Only a few planting materials were from local markets and markets in another place.

### The structure of Salar HGs

Salar houses usually face south for better sunlight and temperature, which is common in northern China. In this way, the houses can catch a cool wind from the south in summer and block the cold wind from the north in winter.

**Fig. 3:**
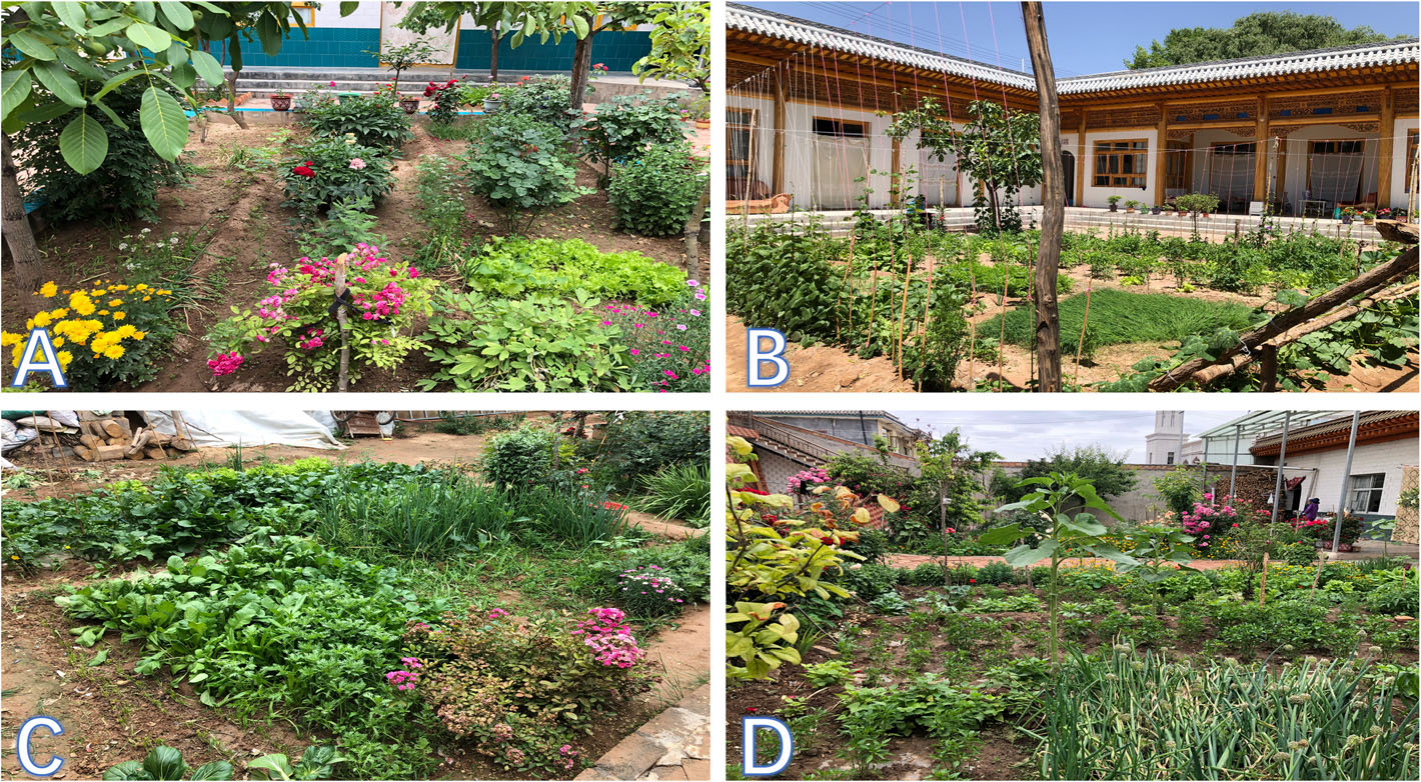
Different HG types

According to the main focuses, the Salar HGs can be classified into different types based on primary production systems, plant composition, and structure of HGs (Fig. 3), including

Ornamental focus (Fig. 3a): HGs with ornamental and fruit trees (27%)

Production focus (Fig. 3b): HGs with vegetables and fruit trees (12%)

Dual-purpose (Fig. 3c): HGs with vegetables and ornamental (11%)

Multi-purpose (Fig. 3d): HGs with multiple planting (51%)

Interestingly, the types of the Salar HGs were related to the householder’s income level. Usually, ornamental plants are more grown in wealthy families, while the production-focused plants are grown more in families with lower income.

The constituent elements of Salar HGs usually include landforms, plant materials, structures, hard elements (like rockery), and water elements. Salar HGs were fenced with bricks, bare soil or cement, and made into fences of different shapes regarding the horizontal structure. We also found many Salar HGs having a unique space in front of the house. Those spaces were usually left bare or covered with grass or planted with few trees for shade. Fruit plantations were usually surrounding the yard and home; vegetable plots were mainly arranged close to the house. Vertically, the Salar HGs can be divided into three levels: the ground level (< 3 m) was grown with vegetables, ornamentals, staple food plants; the upper level (3–10 m) was grown with shrubs like cloves, Chinese prickly ash, and some fruit trees; the highest level was grown with walnut trees or trees for fuelwood. Both arrangements for horizon level and vertical level help make optimal use of the space, soil production capacity, multiple natural resources, and harvest of different crops or fruits for self-sufficiency and other commercial use.

### Agroecosystem services and function of Salar HGs

Salar people have developed an HG system integrated with agriculture, horticulture, aesthetics, and animal husbandry. We found that the Salar HGs, as a typical agroecosystem, possess multiple services and functions that directly benefit households according to the field investigation:

#### Food supplement

Food production as the supplement for Salar’s daily diet is considered the primary function of Salar HGs. However, food production is limited due to the local climate. The Salar HGs can only supply food from April to October each year and provide households with vegetables, fruits, flavors, etc.

#### Occasional income

Usually, the HG products are used for self-sufficiency. We also found that Salar women sell fruits, Chinese prickly ash, pumpkins, and chili peppers grown in HGs to increase their occasional income. According to the interview, the Salar HGs can provide about 150 dollars per year for each household.

#### Small-scale experimentation

**Fig. 4:**
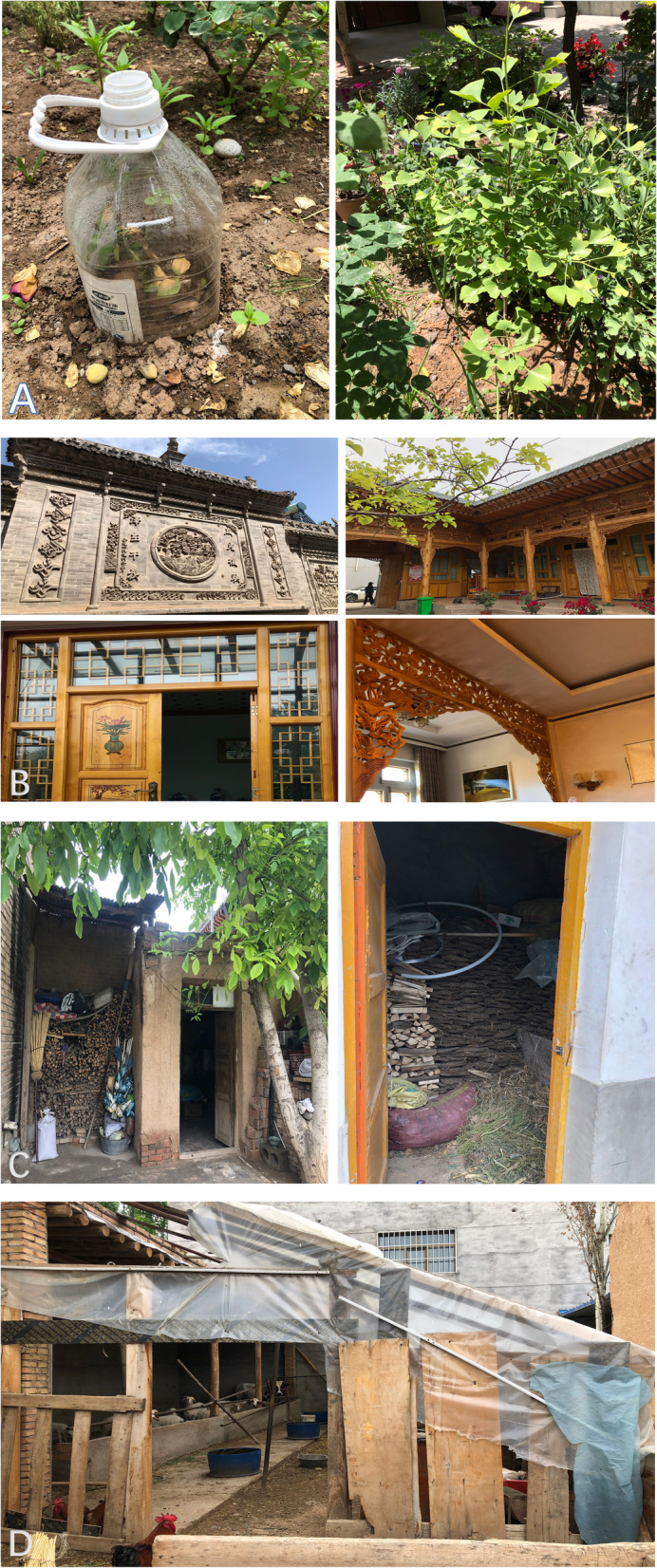
**a** Small-scale experimentation, **b** aesthetic purpose, **c** fuel storage, **d** livestock recommendation

Salar HGs are often utilized as testing plots for new plants and varieties. For instance, some new fruit varieties such as *Diospyros lotus*, *Prunus domestica*, and *Chaenomeles sinensis* from other places or bought from the markets would be cultivated in their HGs for pilot cultivation. The pilot experiment can help screen off the unsuitable plants and make the farming more productive and efficient (Fig. 4a).

#### The agrobiodiversity reservoir

Salar HGs played an important role in biodiversity conservation, especially for local traditional crop varieties. Salar farmers always grow some traditional varieties of crops in their own HGs to conserve the seeds. For example, we recorded 3–5 varieties of *Allium tuberosum*, *Cucumis sativus*, *Zanthoxylum bungeanum*, and *Chrysanthemum morifolium* grown in the Salar HGs. The tradition of exchanging seeds, for Salar people, also contributes to the rich genetic diversity in Salar HGs.

#### Aesthetic purpose

Salar people love ornamentals in their nature. They not only cultivate different ornamental species but also decorate their houses with plant totems. Many plants selected by Salar people for cultivation reflect cultural preferences (Fig. 4b).

#### Fuel storage

There is always a place in Salar HGs to store the fuels, including the fuelwoods collected from the wild and the animal wastes produced in the livestock shed. Although the Salar community uses electricity or coal gas to meet daily life needs, those fuels are still required when having important religious sacrifices and festival celebrations, which are held in Salar HGs (Fig. 4c).

#### Livestock shed

Livestock is an essential part of Salar HGs. Livestock keeping by Salar people mainly provides protein and a source of additional income, primarily from milk, eggs, animal skin, and meat. The Salar ancestors were nomadic people, and they continue to keep the tradition of animal husbandry until now. Almost every household has cattle and sheep (Fig. 4d).

#### Culture service

For Salar people, HGs also play an important role in traditional festival celebrations and religious activities. Salar people’s marriage, funeral, festivals, and religious ceremony must be carried out in their own HGs. The products used for various ceremonies, especially fruits and meat, are used for the festival celebration.

## Discussion

The homegarden is always an essential carrier of traditional knowledge. Only a few cases about homegarden in the Tibet plateau have been reported. Yu et al. also reported a homegarden case in the Tibet plateau, and they found that the plants in local HGs are important sources of plant products such as foods, herbal medicines, and fibers to support daily lives. Comparing with their study result, Salar HGs present some differences. For example, the entertainment and culture functions are one of the main contributions to Salar livelihoods. Also, we hardly found medicinal plants were grown in Salar HGs. One reason might be that Salar people lived in an environment with less biodiversity; they did not accumulate wealthy medicinal knowledge; another reason could be that Salar people are good at doing business brings much income. Thus, they rely more on modern medical treatment instead of traditional herbal treatment.

Our result has described the Salar HG management and presented the plant diversity and agroecosystem services of Salar HGs. It is the first time to report Salar HGs as a scientific paper. As we found, it is a vital medium and carrier to maintain the local Salar culture and related traditional knowledge. However, it also faces a series of challenges in this period with fast urbanization and climate change.

The planting area reduction is one of the biggest challenges. More and more families have owned cars, which give the Salar HGs a new function, car parking, and it also means the reduction of planting area, possibly leading to less biodiversity. Moreover, rebuilding a bigger house also cause the reduction of the planting area of the Salar HGs. Another big challenge is that commercial plants further extruded the living spaces of traditional landraces. On the one hand, some commercial plants are easier to grow and are productive. Thus, they would be preferred by the householders to the traditional landraces. The massive planting of commercial plants is causing the loss of natural genetic resources. On the other side, some commercial plants, especially potted flowers, usually were transported from other parts of China carrying pests and diseases. Naturally, climate change also helped the survival of those pests. Many of our interviewees mentioned that the spread of the pests and diseases in their HGs gave them some economic losses. Many interviewees were citing the situation of a labor shortage. The younger generation likes to go out of the town to seek better income, which would cause the difficulties of traditional knowledge inheritance and daily managing of Salar HGs.

We propose some suggestions based on our research:

1) Better education needed: It is necessary for local Salar people, especially the youngsters, to know how important the Salar HGs are.

2) The devotion of local government: The local government can provide help to protect the local HGs, especially the traditional landraces in it. For example, the local government can invite specialists to guide local farmers to control the pests and plant disease in Salar HGs. Moreover, they can also publish particular policies to encourage local people to plant traditional landraces.

3) Development of local tourisms: The best conservation is to make the Salar HGs sustainably producing benefits for the households economically. Through the development of local tourism and the Salar HGs culture, local people will show more interest to construct and decorate better HGs and provide them with profits so that the HGs and associated traditions can be better conserved.

## Conclusion

This paper reveals the floristic diversity of HGs of Salar communities. The HGs of Salar communities harbor high levels of plant biodiversity on the Qinghai-Tibet Plateau. According to primary production systems, there are 4 different types of HGs, including ornamental focus, product focus, dual-purpose, and multi-purpose. In total, 108 plant species (excluding weeds and bonsai) within 43 families were recorded from Salar HGs in the study area. The most important and frequent plants are *Rosa chinensis, Prunus salicina*, and *Ziziphus jujuba.* The average number of plants varied from 4 to 32 species in each homegarden in three investigated townships. We found that the Salar HGs, as a typical agroecosyste,prossess multiple servcices and functions that directly benefit households according to the field investigation. Ecosystem services and function research suggested that Salar homegarden agroecosystems provides services mainly related to supply and culture services. In summary, Salar HGs are important to provide food supplements, aesthetics, and cultural spaces where knowledge about agricultural practices is transmitted. It is necessary to learn and protect the traditional knowledge associated with the HGs for agrobiodiversity conservation and rural livelihood.

## Data Availability

Raw and treated data generated during the study are available from the corresponding author on reasonable request.
